# Cortical signature of depressive symptoms in frontotemporal dementia: A surface‐based analysis

**DOI:** 10.1002/acn3.51860

**Published:** 2023-07-31

**Authors:** Alma Ghirelli, Benedetta Tafuri, Daniele Urso, Giammarco Milella, Roberto De Blasi, Salvatore Nigro, Giancarlo Logroscino, for the Frontotemporal Lobar Degeneration Neuroimaging Initiative

**Affiliations:** ^1^ Center for Neurodegenerative Diseases and the Aging Brain, Department of Clinical Research in Neurology University of Bari ‘Aldo Moro’, “Pia Fondazione Cardinale G. Panico” Lecce Italy; ^2^ Department of Translational Biomedicine and Neuroscience (DiBraiN) University of Bari ‘Aldo Moro’ Bari Italy; ^3^ Department of Neurosciences, King's College London Institute of Psychiatry, Psychology and Neuroscience London UK; ^4^ Department of Diagnostic Imaging Pia Fondazione di Culto e Religione “Card. G. Panico” Lecce Italy; ^5^ Institute of Nanotechnology (NANOTEC), National Research Council Lecce Italy

## Abstract

**Background and Objectives:**

Depressive symptoms are frequently reported in patients affected by frontotemporal dementia (FTD). At structural MRI, cortical features of depressed FTD patients have been poorly described. Our objective was to investigate correlations between cortical measures and depression severity in FTD patients.

**Methods:**

Data were obtained from the Frontotemporal Lobar Degeneration Neuroimaging Initiative (FTLDNI) database. We included 98 controls and 92 FTD patients, *n* = 38 behavioral variant FTD (bvFTD), *n* = 26 non‐fluent variant Primary Progressive Aphasia (nfvPPA), and *n* = 28 semantic variant Primary Progressive Aphasia (svPPA). Patients underwent clinical and cognitive evaluations, as well as a 3D T1‐weighted MRI on a 3 Tesla scanner (Siemens, Trio Tim system). Depression was evaluated by means of Geriatric Depression Scale (GDS). Surface‐based analysis was performed on T1‐weighted images to evaluate cortical thickness, a measure of gray matter integrity, and local gyrification index (lGI), a quantitative metric of cortical folding.

**Results:**

Patients affected by svPPA were more depressed than controls at NPI and depression severity at GDS was higher in svPPA and bvFTD. Severity of depression correlated with a decrease in lGI in left precentral and superior frontal gyrus, supramarginal and postcentral gyrus and right precentral, supramarginal, superior parietal and superior frontal gyri. Furthermore, depression severity correlated positively with cortical thickness in the left medial orbitofrontal cortex.

**Discussion:**

We found that lGI was associated with depressive symptoms over brain regions involved in the pathophysiology of major depressive disorder. This finding provides novel insights into the mechanisms underlying psychiatric symptoms in FTD.

## Introduction

The term frontotemporal dementia (FTD) encompasses a wide range of clinical syndromes, which are classified into three different subtypes based on symptoms, neuropsychological and neuropsychiatric profiles as well as patterns of atrophy at magnetic resonance imaging (MRI).^1^ These include behavioral variant FTD (bvFTD),^2^ semantic variant primary progressive aphasia (svPPA, or semantic dementia SD), and non‐fluent variant PPA (nfvPPA).^3^ Neuropathologically, FTD can be characterized by the accumulation of different aggregates, such as TAR DNA‐binding protein 43 (TDP‐43) in 50% of cases, microtubule‐associated protein tau (MAPT) in 40% of cases, and fused in sarcoma (FUS) in another 5%–10% of cases.^4^

Neuropsychiatric symptoms such as depression and apathy are frequently reported in all FTD subgroups and represent a substantial burden for both patients and caregivers.^5^ In a recent systematic review^6^ depression prevalence ranged between 7.7% and 69.6% in FTD patients. More in detail, across three PPA studies, prevalence of depression was reported between 38.2% and 43%.^7^, ^8^, ^9^ For what concerns bvFTD instead, most studies found depression prevalence falling between 22% and 52%.^7^, ^10^

In the past years, several efforts have been made in identifying pathological and imaging features associated with neuropsychiatric symptoms (including disinhibition, apathy, perseverative/compulsive behaviors, hyperorality, depression, hallucination, mania, and delusions) in FTD patients.^11^, ^12^, ^13^, ^14^, ^15^ What emerged from a cohort of 103 brain donors with a frontotemporal lobar degeneration (FTLD) pathology was that hyperorality could best distinguish underlying FTLD from non‐FTLD pathology, while the presence of auditory hallucinations was associated with FTLD‐TDP pathology, and perseverative or compulsive behavior pointed to FTD‐TDP type B or C histotypes. However, no specific pattern of pathology was associated to depression or apathy symptoms.^11^ Concerning neuroimaging investigations, only a few MRI studies have focused on the neural correlates of psychiatric manifestations in patients with FTD.^12^, ^13^, ^14^, ^15^ A predominant right‐sided atrophy at the level of some frontal, temporal and parietal cortical structures was observed in FTD patients affected by apathy or anhedonia.^13^, ^14^, ^15^ Increased cortical thickness was also reported in FTD patients with depression compared to not depressed patients, primarily in right fronto‐insular cortical areas.^13^

In recent years, several studies have shown that the examination of cortical folding could provide interesting information on gray matter (GM) alterations associated with cognitive functions in normal and pathological conditions.^16^, ^17^, ^18^, ^19^ Local gyrification index (lGI) is the most common measure of cortical folding able to quantify the amount of cortical surface invaginated in the sulci and to identify and localize minor gyral anomalies.^16^ Cortical folding has been adopted to pick cortical abnormalities in early‐stage Alzheimer's disease,^20^ in presymptomatic C9orf72 expansion carriers^17^ as well as to monitor cortical progression of pathology in Parkinson's disease.^18^ Cortical folding has also been used to differentiate the patterns of cortical changes in Alzheimer's disease and FTD.^19^ Gyrification index has been widely used to map areas of cortical alteration in several psychiatric disorders, presumably reflecting their neurodevelopmental etiology, including major depressive disorder,^9^, ^21^, ^22^ borderline personality disorder,^22^ schizophrenia,^23^ bipolar disorder,^24^ and autism.^9^

To date, no study has adopted the gyrification index to examine the cortical neurobiology of depressive symptoms in FTD patients. Considering the evidence of gyrification abnormalities in primary depressive syndromes, we hypothesized that GM measures would result altered in regions that play a key role in the pathophysiology of depression such as cingulate, orbitofrontal, dorsolateral, and ventrolateral prefrontal cortices.

## Methods

### Participants

Data used in the current study were obtained from the Frontotemporal Lobar Degeneration Neuroimaging Initiative (FTLDNI) database. The FTLDNI was funded through the National Institute of Aging and started in 2010. The primary goals of FTLDNI are to identify neuroimaging modalities and methods of analysis for tracking frontotemporal lobar degeneration and to assess the value of imaging versus other biomarkers in diagnostic roles. The principal investigator of NIFD was Dr. Howard Rosen, MD at the University of California, San Francisco. The project is the result of collaborative efforts at three different sites in North America. For up‐to‐date information on participation and protocol, please visit: http://memory.ucsf.edu/research. Data were downloaded through the LONI platform after approved by the data access committee. We included 98 healthy controls (HC), 38 patients with bvFTD,^2^ 26 patients with nfvPPA, and 28 svPPA patients^3^ with a valid baseline T1‐weighted MRI. Controls were included if they had a normal neurologic assessment, Mini‐Mental State Examination score ≥ 28, and no family history of neurodegenerative diseases. Patients were excluded if they had medical illnesses, a history of substance abuse or if they presented with major brain lesions (vascular, other). Of note, we considered only participants scanned at University of California, San Francisco (UCSF), the largest recruiting center, to avoid potential bias due to different scanners and acquisition imaging protocol. All patients underwent neurological examination, multi‐domain cognitive testing, and brain MRI at study entry.

### Standard protocol approvals, registrations, and patient consents

Approval for the FTLDNI protocol has been granted by institutional review board at the study site.

### Neuropsychological assessment

Global cognition was evaluated by means of the Mini‐Mental State Examination (MMSE), while disease severity was scored according to the Clinical Dementia Rating Scale. Scores of CDR sum of boxes (CDR‐SB) were recorded as well. Neuropsychological assessment comprised the form of Neuropsychiatric Inventory (NPI‐Q ratings)^25^ and the 30‐item Geriatric Depression Scale (GDS‐30).^26^ This scale has been successfully used to characterize depressive symptoms in several neurodegenerative conditions including PPA and bvFTD and has been shown to be sensitive to depression in post‐stroke patients with and without aphasia.^27^, ^28^ Based on the response to GDS‐30 we identified as depressed subjects scoring ≥10. Furthermore, the presence and severity of apathy and depression have been recorded as part of the NPI.

### MRI acquisition and processing

MR images were acquired on a 3T Siemens Trio Tim system equipped with a 12‐channel head coil at the UCSF Neuroscience Imaging Center. Structural images were acquired using a T1‐weighted MPRAGE (TR/TE = 2300/2.9 ms, matrix = 240 × 256 × 160, isotropic voxels 1 mm^3^, slice thickness = 1 mm). All T1‐weighted scans were visually inspected to recognize indications of scan artifacts and motion. For surface‐based analyses of cortical thickness and gyrification, we applied the automated surface‐preprocessing algorithms implemented in the CAT12 toolbox that allow the simultaneous estimation of cortical thickness and the reconstruction of the central surface of the left and right hemisphere by using the projection‐based thickness method.^29^ This methodological approach represents an accurate and robust solution for cortical estimation, representing a reliable and fast alternative to FreeSurfer.^30^, ^31^, ^32^ Using a tissue segmentation to estimate the white matter (WM) distance, the software projects the local maxima (which is equal to the cortical thickness) to other GM voxels by using a neighbor relationship described by the WM distance. This approach includes partial volume correction, correction for sulcal blurring and for sulcal asymmetries. Prior to analyses, the left and right hemisphere of each participant surface‐based cortical thickness data were merged and smoothed with a 15 mm FWHM isotropic Gaussian kernel as recommended by the authors of the CAT12 toolbox. Similarly, CAT12 extracted cortical gyrification values at local (vertex) level, based on a spherical harmonic reconstruction method described in previous studies.^33^, ^34^ After obtaining individual vertex‐wise gyrification maps, smoothing was performed with a 20 mm FWHM kernel as recommended. Finally, after the pipeline run, the outputs of the CAT12 structural pipelines were visually checked for reconstruction cortical surface.

### Statistical analysis

Normality of the data were tested using the Shapiro–Wilk test. Variables with normal distribution were compared across groups using ANOVA followed by pairwise *t*‐tests. Non‐normally distributed variables, instead, were compared across groups using Kruskal–Wallis test, followed by pairwise Mann–Whitney U test. Categorical variables were compared with chi‐squared test and partial correlations. Analysis was performed with IBM SPSS Statistics 23 (IBM Corporation, New York, EUA). Statistical significance for all tests was set at *p* < 0.05 and all *p*‐values were corrected for multiple comparisons (Bonferroni).

Concerning imaging analyses, GM cortical changes between FTD patients and controls and associations between surface‐based properties and GDS scores were investigated using a vertex‐based approach. In particular, whole‐brain statistical analyses were performed using the threshold‐free cluster enhancement (TFCE) toolbox (dbm.neuro.uni‐jena.de). This nonparametric permutation‐based approach was performed with 5000 permutations using a significant statistical threshold of family‐wise error rate (FWE)‐corrected *p* < 0.05. Vertex‐wise analyses comparing controls and FTD patients were corrected for age, gender, education, and MMSE score. Correlation analyses in the FTD group were corrected for age, gender, education, CDR‐SB, MMSE, and apathy.

## Results

### Sociodemographic and clinical features

Demographic and clinical characteristics of FTD patients are summarized in Table 1. bvFTD patients had a lower level of education compared to controls (*p* < 0.001), while nfvPPA patients were the eldest compared to both bvFTD, svPPA patients and controls (*p* < 0.001). In terms of cognition, patients had lower MMSE compared to controls. bvFTD patients scored higher in terms of CDR and CDR‐SB compared to nfvPPA and svPPA (*p* < 0.001). Furthermore, GDS was not associated to disease severity as measured by CDR (*p* = 0.484) and CDR‐SB (*p* = 0.299).

**Table 1:** Socio‐demographic and clinical characteristics of healthy controls, bvFTD, nfvPPA, and svPPA patients.

Subjects	HC	bvFTD	nfvPPA	svPPA	*p*
Number	98	38	26	28	–
Sex (M/F)	45/53	24/14	12/14	14/14	0.333
Education (years) (*N*)	17.4 ± 1.8 [12–20] (98)	15.3 ± 2.6 [10–20] (38)*	16.2 ± 2.5 [12–20] (26)	16.4 ± 2.6 [10–20] (28)	**<0.001**
Age at MRI (years) (*N*)	63.2 ± 7.3 [36–81] (98)	60 ± 6.8 [46–74] (38)	68.9 ± 8.0 [54–81] (26)* +	62.5 ± 6.3 [50–72] (28)$	**<0.001**
MMSE (*N*)	29.4 ± 0.8 [27–30] (98)	24.7 ± 4.2 [12–30] (38)*	26.4 ± 3 [18–30] (26)*	25.8 ± 3.5 [15–30] (28)*	**<0.001**
CDR (*N*)	–	1.09 ± 0.53 [0–2] (38)	0.4 ± 0.32 [0–1] (25)+	0.64 ± 0.32 [0.5–2] (28)+	**<0.001**
CDR‐sb (*N*)	–	6.1 ± 2.7 [0–13] (38)	1.7 ± 1.7 [0–5.5] (25)+	3.4 ± 1.8 [1–9.5] (28)+ $	**<0.001**
Depression (No/Yes) (*N*)	84/14 (14.2%) (98)	25/13 (34.2%) (38)	21/5 (19.2%) (26)	16/12 (46.2%) (26)*	**0.004**
Depression NPI (%) (No/Yes)	51/5 (8.9%) (56)	28/8 (22.2%) (36)	17/7 (29.1%) (24)	15/13 (46.4%) (28)*	**0.001**
Depression severity (GDS 30)	3.9 ± 4.4 [0–20] (98)	7.1 ± 6.6 [0–24] (38)*	6.5 ± 4.8 [0–20] (26)	8.9 ± 5.6 [1–25] (28)* +	**0.001**
Apathy NPI (%) (No/Yes) (*N*)	55/1 (1.8%) (56)	2/34 (94.4%) (36)*	13/11 (45.8%) (24)* +	5/23 (82.1%) (28)* $	**<0.001**

Values are reported as means ± standard deviations [min. value – max. value]. *N* represents the number of patients with that available data. *p* values refer to Pearson chi‐squared ANOVA tests with Bonferroni post hoc analysis. Depression is defined by a score of ≥ 10 at GDS30. Depression and apathy are further assessed by means of the NPI. Bold values represent significant results. Symbols: * = significantly different from HC; + = significantly different from bvFTD; $ = significantly different from nfvPPA.

### Neuropsychological features

Patients with svPPA (*p* = 0.005) and bvFTD (*p* = 0.045) were more depressed than controls by means of number of depressed patients. Depression severity both at GDS and at NPI was higher in svPPA (*p* < 0.001) compared to controls. Apathy, instead, was more prevalent in nfvPPA (82.1%) and bvFTD (94.4%) patients compared to other groups (*p* < 0.001).

### Gray matter cortical differences between FTD patients and controls

**Figure 1:**
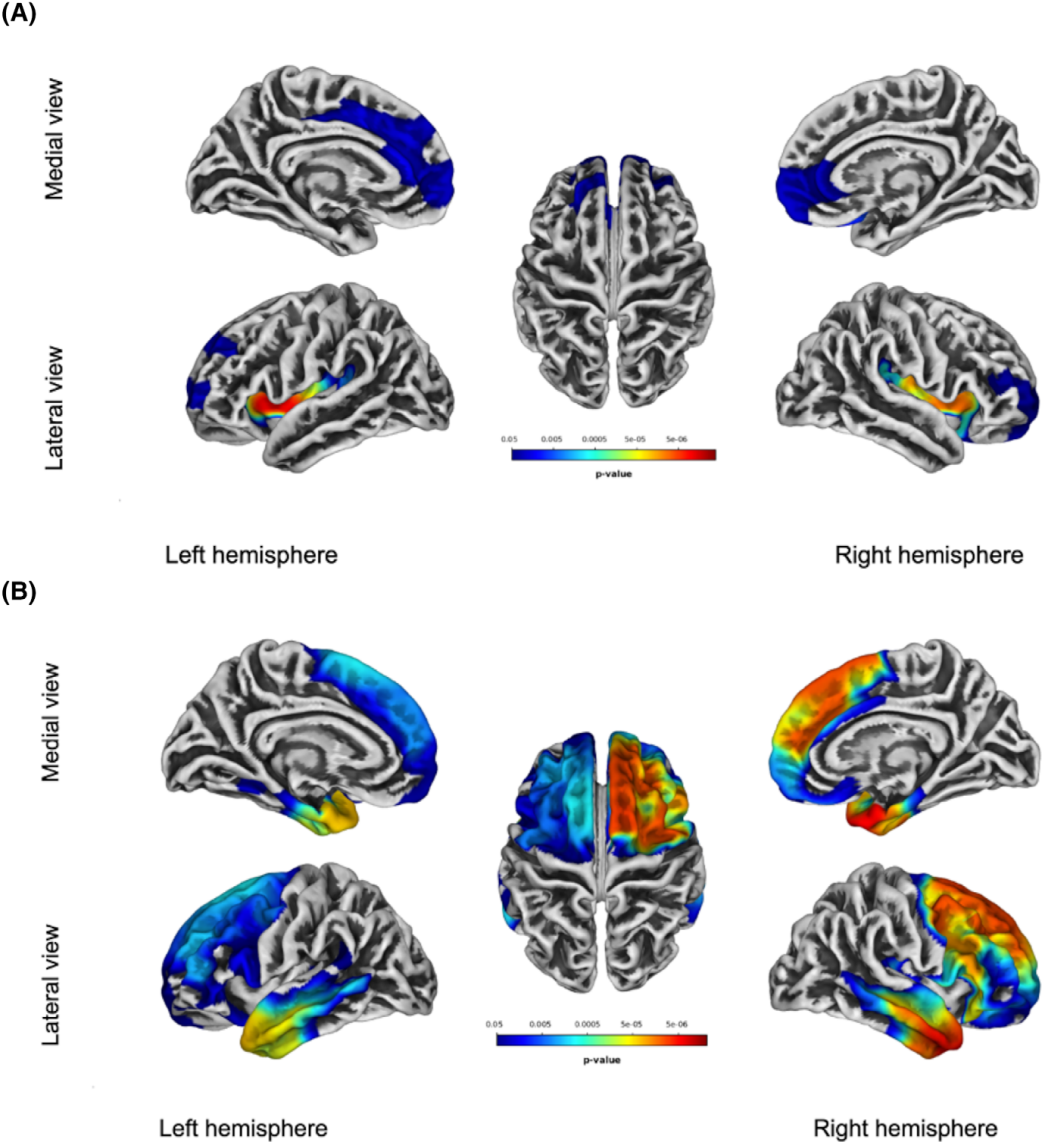
Comparison between controls and FTD patients. Patients with FTD showed a reduced gyrification (A) and cortical thickness (B) in frontotemporal brain regions. Results are FWE‐corrected.

Compared with controls, patients with FTD showed a reduced gyrification in frontal and temporal cortical areas including the superior frontal cortex, insula, superior temporal gyrus and the medial and lateral regions of the orbitofrontal cortex (Fig. 1, upper panel). A more widespread pattern of cortical thickness decrease was also found between FTD and control groups (Fig. 1, lower panel).

### Association between gyrification and GDS in FTD and controls

**Figure 2:**
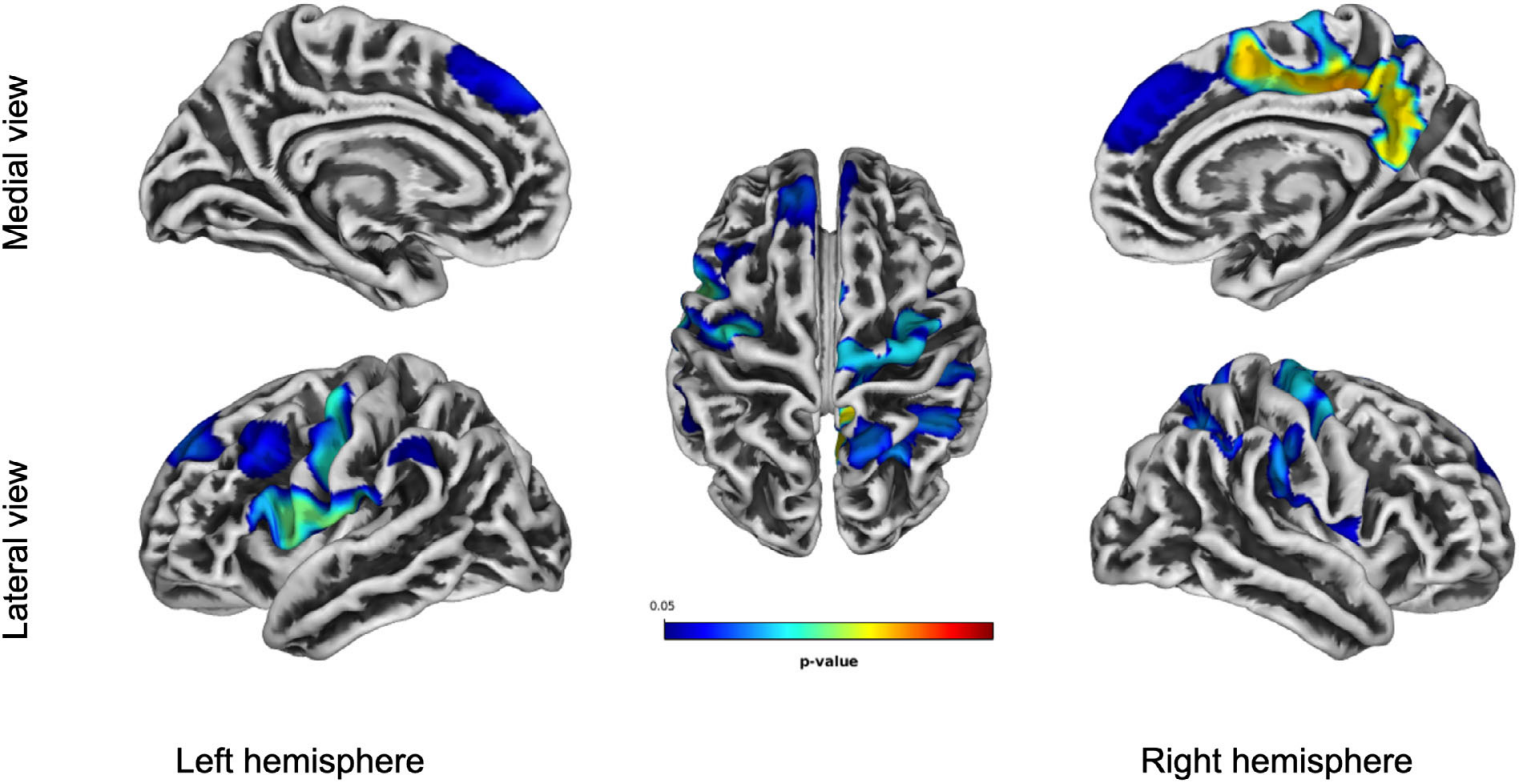
Correlation between GDS30 and local gyrification index (lGI). Depression severity correlates with a decreased lGI at the level of the left frontotemporal cortex and of the right fronto‐parietal cortex. Results are FWE‐corrected. Legend: red represents stronger correlation.

Figure 2 and Table 2 summarize the correlation between lGI values and GDS30 scores. Severity of depression correlated with a decreased lGI at the level of left frontotemporal cortices, mainly in the precentral, superior, and middle frontal gyrus, postcentral gyrus, as well as in the pars opercularis, middle frontal, and rostral middle frontal gyrus (*p* < 0.05, FWE‐corrected). On the right hemisphere, the precentral, supramarginal, superior parietal, and precuneus were the more strictly correlated to depression severity, as well as superior frontal, paracentral and post‐central gyrus (*p* = 0.01, FWE‐corrected). Correlation analysis between gyrification values and depression severity were also performed in different FTD subgroups showing that no specific disease phenotype drove the results obtained in the whole group (Fig. S1). No statistically significant association emerged between gyrification and GDS30 in controls.

**Table 2:** Significant correlations between GDS30 and local gyrification index (lGI) in patients with frontotemporal dementia.

Local gyrification index (lGI)			
*p*‐value (FWE‐corrected)	Size	Overlap of atlas region	
*Negative correlation‐Left hemisphere*			
0.0253	760	46%	superior frontal
		30%	caudal middle frontal
		24%	rostral middle frontal
0.0324	736	38%	precentral
		32%	pars opercularis
		23%	postcentral
		7%	supramarginal
0.0439	278	99%	precentral
*Negative correlation‐Right hemisphere*			
0.0069	4332	19%	superior parietal
		17%	precuneus
		17%	precentral
		14%	superior frontal
		12%	paracentral
		8%	inferior parietal
		6%	supramarginal
		4%	posterior cingulate
		1%	isthmus cingulate
		1%	postcentral
0.0334	1239	48%	supramarginal
		30%	precentral
		17%	postcentral
		3%	pars opercularis
		2%	caudal middle frontal

### Association between cortical thickness and GDS in FTD patients and controls

**Figure 3:**
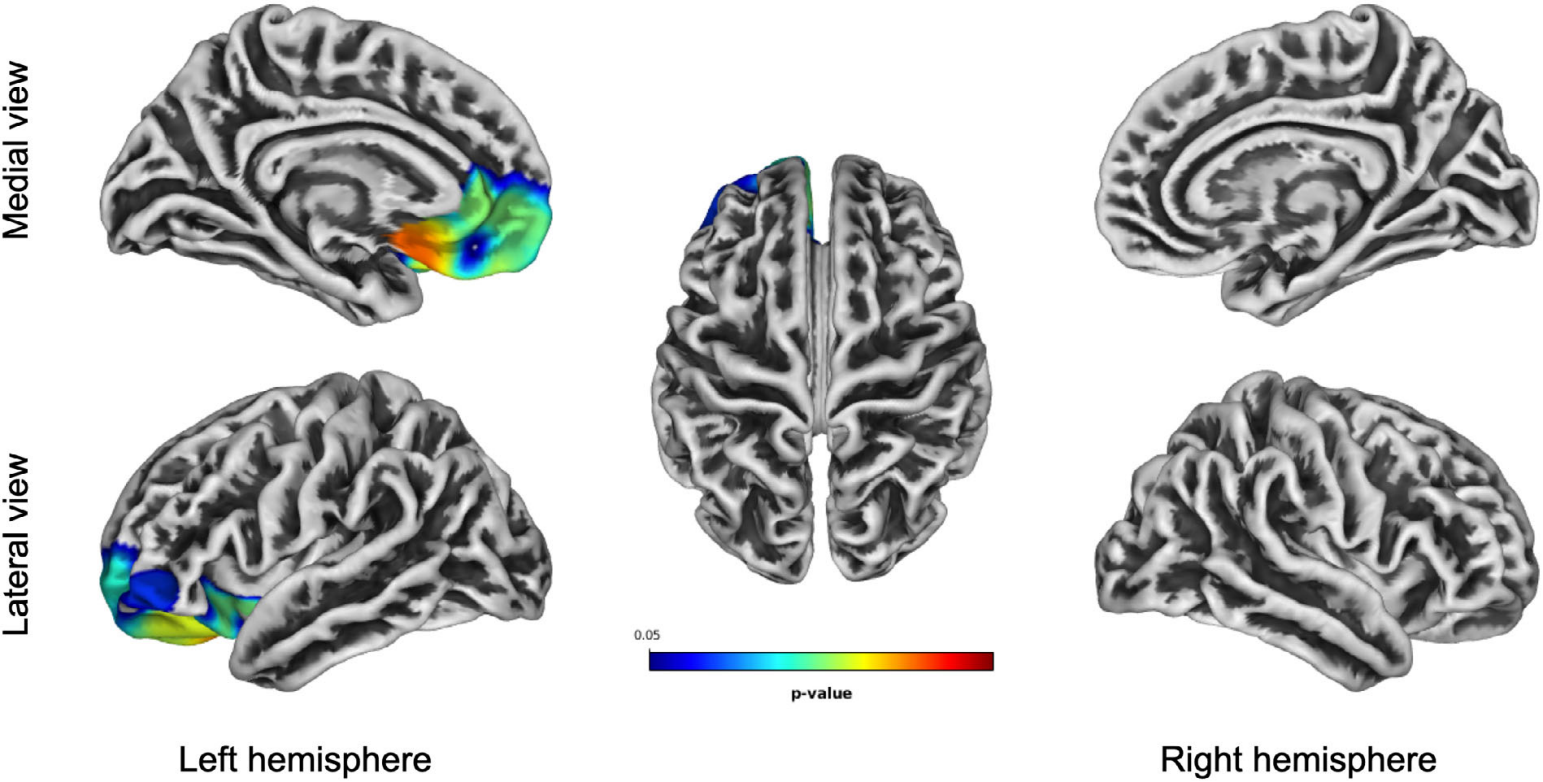
Correlation between GDS and cortical thickness. Increased depression correlates with increased cortical thickness at the level of the left medial orbitofrontal cortex. Results are FWE‐corrected. Legend: red represents stronger correlation.

Figure 3 and Table 3 summarize the correlation between cortical thickness and GDS30. Severity of depression correlated positively with cortical thickness mainly at the level of the left medial orbitofrontal cortex (*p* < 0.05, FWE‐corrected). No significant correlations were found between cortical thickness values and GDS30 in controls.

**Table 3:** Significant correlations between GDS30 and cortical thickness in patients with frontotemporal dementia.

Cortical thickness			
*p*‐value (FWE‐corrected)	Size	Overlap of atlas region	
*Positive correlation‐Left hemisphere*			
0.0094	2298	33%	lateral orbitofrontal
		22%	medial orbitofrontal
		13%	rostral middle frontal
		10%	rostral anterior cingulate
		8%	superior frontal
		5%	insula
		4%	pars orbitalis
		2%	frontal pole

## Discussion

In this study, surface‐based morphometry analysis was used to investigate correlations between GM cortical changes and symptoms of depression in FTD patients. In our work, higher GDS30 scores were associated with a reduction of lGI at the level of precentral and supramarginal gyri bilaterally, left superior frontal gyrus and pars opercularis, as well as right superior parietal and superior frontal gyri. A positive association between GDS30 and cortical thickness in the left medial orbitofrontal cortex was also found in FTD patients.

Overall, local gyrification values were associated to depression severity in cortical brain regions that play a key role in the pathophysiology of major depressive disorder such as the cingulate cortex, orbitofrontal cortex, dorsolateral and ventrolateral prefrontal cortices.^9^, ^35^ Volume loss in frontal regions has also been recognized as the most common finding in major depressive disorder (MDD), especially at the level of the orbitofrontal cortex, middle prefrontal cortex and dorsolateral prefrontal cortex.^9^, ^36^, ^37^ Furthermore, it has been previously demonstrated that thickness reduction in prefrontal areas is associated to MDD and that these changes are associated with poor clinical outcomes.^9^, ^21^, ^35^ A reduction in lGI at the level of the left insula/frontal operculum bilaterally, left medial orbitofrontal cortex, and the left cingulate was also reported in patients affected by MDD compared to controls.^38^ Not surprisingly, the same areas involved in the circuit of depression in MDD were altered in our analysis, suggesting that depressive symptoms carry a cortical footprint that might be reproduced in different clinical conditions. Indeed, several similarities can be found in GM changes associated to depression across neurodegenerative diseases.^39^ Abnormal metabolic activity of the anterior cingulate gyrus was recognized in both MDD and depressed Alzheimer's disease (AD) patients, but it was not found in non‐depressed AD patients.^40^ In Parkinson's disease, current or prior history of MDD was associated with cortical thinning of frontotemporal lobes and anterior cingulate cortex,^41^ while minor depression was associated to hypoperfusion in the upper and lower left frontal gyri.^42^

When looking at the correlation between GDS and cortical thickness, a higher thickness in ventromedial prefrontal cortex (vmPFC) was positively associated with depressive symptoms. Previous studies in FTD and AD patients disclosed a similar association between cortical thickness in anterior cingulate, ventromedial and inferior frontal cortex and depression symptoms.^13^, ^43^ Taken together, these findings seem to suggest that a preserved cortical thickness in frontal brain regions involved in emotional information processing may represent a key requisite to experience depressive symptoms.

Our study demonstrates that changes in gyrification are not attributable to a decrease in cortical thickness and that cortical folding as measured by lGI is more valuable at identifying differences at GDS. This is in line with other studies that have correlated different GM metrics to lGI. Gyrification, indeed, has been demonstrated to be correlated with GM volume and not cortical thickness.^44^ Furthermore, it has been demonstrated to relate to WM connectivity and WM arrangement rather than to cortical thickness.^45^ This should not be surprising given that cortical thickness and lGI have different developmental trajectories and reflect diverse cellular mechanisms.^46^, ^47^ Indeed, cortical thickness reflects the number of horizontal layers in the cortical columns, including neurons and neuropil. On the other hand, lGI reflects the microstructure of neuronal sheets and summarizes the folding pattern of the brain at a local level, being also associated with local axonal connectivity.^48^ This suggests that gyrification, notwithstanding the fact that is a surface measure, carries a functional meaning that oversteps mere morphology.

Whether the loss of gyrification could be explained by a neurodevelopmental hypothesis rather than being part of the neurodegenerative process remains unsolved. Several studies have documented that cortical development may contribute to the neuropathology of major depressive disorder,^49^, ^50^ raising the doubt that at least part of the depressive symptomatology could be attributed to cortical anomalies at development. Indeed, cortical folding could be explained by two different theories, based on a tension‐based hypothesis and a convolutional hypothesis, respectively.^51^, ^52^ According to the former, the main drivers of cortical folding are tensive forces that are applied on the cortical surface; on the other hand, convolutional hypothesis is based on the idea that what drives cortical folding are the different rates of growth of cortical layers. Local gyrification index, which is a metric of both cortical folding and cortical width, can be therefore a good measure of early neural development. The decreased lGI in cingulate, orbitofrontal and frontal cortex in depressed FTD patients might be explained by a disruption of these folding mechanism during neural development, which would result in disruption of mood regulating circuits.

Interestingly, in our cohort of FTD patients, svPPA and bvFTD patients were more depressed compared to controls and depression severity was more significant in svPPA compared to controls. Other studies have reported that in FTD subgroups, the frequency of depressive symptoms reached the highest among svPPA patients (44%–78%).^9^, ^53^ Isolated deficits in language comprehension may indeed induce a person to depression and social withdrawal, which may explain the elevated number of depressed patients among those affected by svPPA. Moreover, a study on PPA patients evidenced that GDS scores in PPA patients was on average higher compared to controls and that those patients were also more likely (43%) than the non‐depressed group (15%) to have a premorbid history of depression.^9^

The current study has some limitations which have to be pointed out. First, the cross‐sectional design of the study does not allow to follow the evolution of depressive symptomatology while the disease unfolds and to correlate it with a deterioration of cortical anomalies. Second, no information was available about the onset of depressive symptomatology or whether patients have been initiated on antidepressive medications. This renders more difficult to speculate on whether the pattern of loss of gyrification could have explained some depressive symptoms even before disease onset. Third, comprehensive neuropsychological information was not available for all FTD patients. Nonetheless, to account for a possible effect of cognitive impairments on depression symptoms all correlation analyses were corrected for MMSE. Finally, genetic data were not available which, therefore, does not allow us to directly compare our findings on the association between gyrification and depression with previous studies on neurodevelopmental effects in genetic forms of FTD.

To the best of our knowledge, this was the first study to adopt a measure of cortical complexity such as gyrification to study the presence and severity of depressive symptoms in a cohort of FTD patients. We demonstrated that local gyrification values were associated to depression severity in cortical brain regions that play a key role in the pathophysiology of major depressive disorder. Further longitudinal studies are warranted to map neuropsychiatric symptoms to structural and functional brain properties in FTD. Finally, investigating brain alterations in a presymptomatic cohort could shed light on the intriguing neurodevelopmental hypothesis in FTD.

## Author Contributions

Alma Ghirelli contributed to the study concept, design, analysis, interpretation of data, and drafting of the manuscript. Salvatore Nigro and Daniele Urso also contributed to the study concept, analysis, interpretation of data and drafting of the manuscript. Benedetta Tafuri contributed to the study concept, analysis, and interpretation of data. Giammarco Milella contributed to the study design and interpretation of data. Roberto De Blasi was responsible for acquisition of data and revision of the manuscript. Corresponding author Giancarlo Logroscino was responsible for overall supervising in acquisition, interpretation of data and revision of the manuscript.

## Funding Information

This work has been supported with the funding of Regione Puglia and CNR for Tecnopolo per la Medicina di Precisione. D.G.R. n. 2117 of 21.11.2018 (CUPB84I18000540002)—C.I.R.E.M.I.C. (Research Center of Excellence for Neurodegenerative Diseases and Brain Aging)—University of Bari “Aldo Moro”.

## Conflict of Interest

Alma Ghirelli, Benedetta Tafuri, Dasniele Urso, Giammarco Milella, Roberto De Blasi, Salvatore Nigro, and Giancarlo Logroscino have no conflicts of interest to declare.

## Supporting information


Supplemental Figure 1.

Data S1.

## Data Availability

The dataset used and analyzed for the current study are available from the corresponding author on reasonable request.
