# Supplementary figures and images for: Cortical signature of depressive symptoms in frontotemporal dementia: A surface‐based analysis
Source: Ann Clin Transl Neurol. 2023 Jul 31;10(10):1704–13. doi: 10.1002/acn3.51860 (PMC10578898; doi:10.1002/acn3.51860)

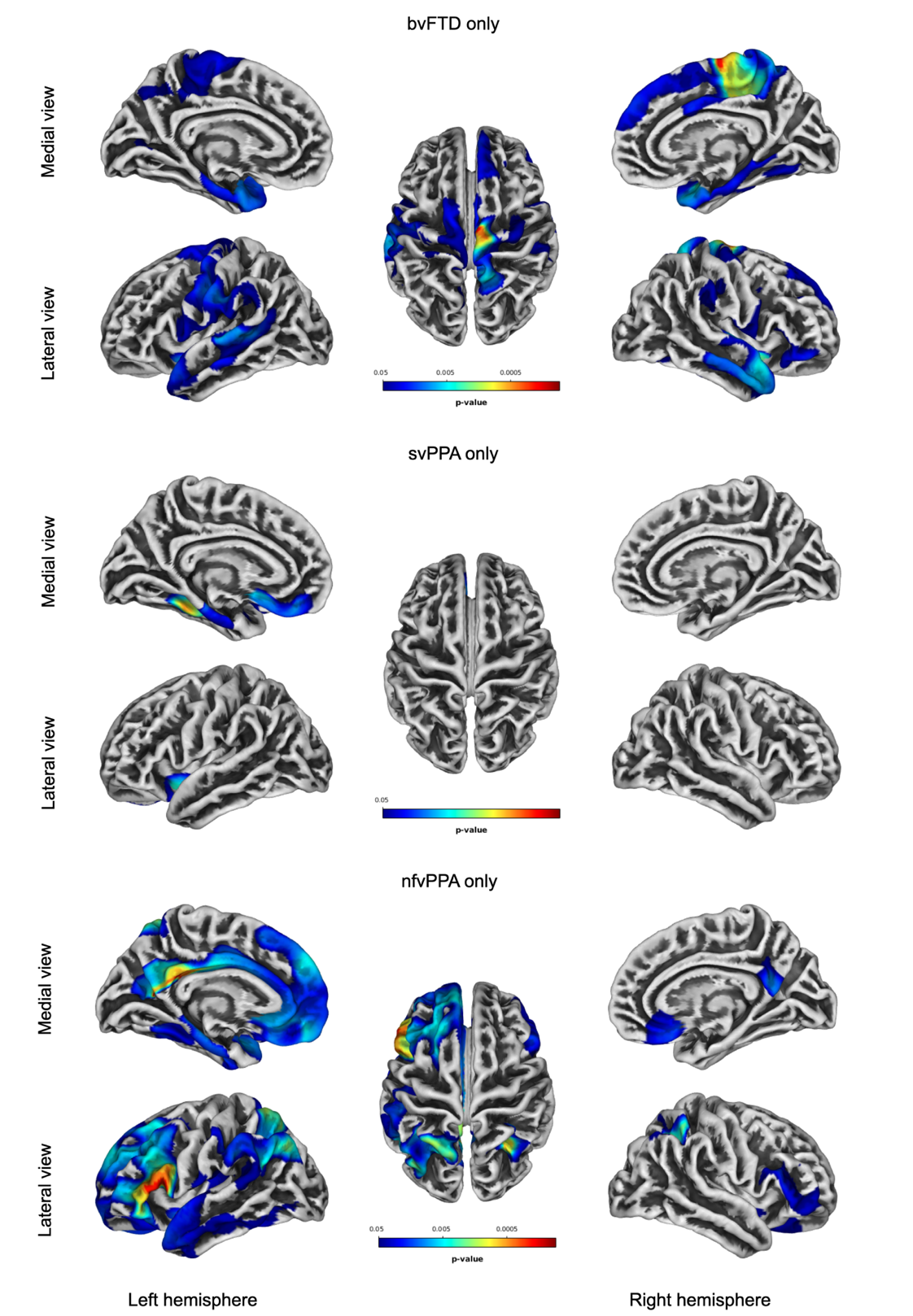

Supplement: Supplementary file 1 — Supplemental Figure 1. [file ACN3-10-1704-s001.png]
