# Supplementary material for: Cortical signature of depressive symptoms in frontotemporal dementia: A surface‐based analysis
Source: Ann Clin Transl Neurol. 2023 Jul 31;10(10):1704–13. doi: 10.1002/acn3.51860 (PMC10578898; doi:10.1002/acn3.51860)
Supplement: Supplementary file 2 — Data S1. [file ACN3-10-1704-s002.docx]

**Supplemental Figure 1. Correlation between GDS and local gyrification index in FTD subgroups.** In each FTD subtype depression severity correlates with a decreased lGI at the level of the left fronto-temporal cortex and of the right fronto-parietal cortex. Results are uncorrected (p<0.05). Legend: red represents stronger correlation.
